# *Ex vivo* Evaluation of the Dynamic Morphometry of the Caudal Cervical Intervertebral Disc Spaces of Small Dogs and Cats

**DOI:** 10.3389/fvets.2021.706452

**Published:** 2021-08-16

**Authors:** Sebastian C. Knell, Lucas A. Smolders, Antonio Pozzi

**Affiliations:** Clinic for Small Animal Surgery, Vetsuisse Faculty University of Zurich, Zurich, Switzerland

**Keywords:** caudal cervical spondylomyelopathy, wobblers disease, morphometry, pathogenesis, intervertebral disc space

## Abstract

The objective of this study was to provide a morphometric description of the caudal cervical intervertebral disc (IVD) spaces of small-breed dogs and cats. Specimens consisting of C4 through C7 from five small-breed dogs and six cats were positioned in neutral, flexion, extension, and lateral bending positions; and CT images were acquired. Height and width of the cranial and caudal vertebral endplates (VEPs), angle between the VEPs (IVD wedge angle), and craniocaudal distance (IVD width) between VEPs for the four loading positions were measured and compared for three segments (C4–C5, C5–C6, and C6–C7). VEP size normalized to body weight from medium-sized dogs was retrieved from a previous study and compared with data from small dogs and cats. A linear mixed model was used to compare outcome measures. Significance was set to *p* < 0.05. VEP size normalized to body weight was the largest in small dogs compared with cats (*p* = 0.0422) and medium-sized dogs (*p* = 0.0064). Cats and medium-sized dogs were similar (*p* = 0.2763) in this regard. Flexion and extension induced a reduction of IVD width in the ventral portion of the IVD and the area of the nucleus. The dorsal part of the IVD remained unchanged throughout loading conditions. Unique morphometric characteristics of the caudal cervical IVD space of small dogs and cats were detected that are different from those described in sizes of dogs (medium-sized) typically affected by caudal cervical spondylomyelopathy (CSM). These findings may help to understand the different pathomechanisms in cervical spinal disease between small- and medium-sized dogs, including caudal CSM.

## Introduction

Caudal cervical spondylomyelopathy (CSM) is a common disease in middle- to large-breed dogs involving neurological signs resulting from compression of the spinal cord and the nerve roots in the caudal cervical spine (C5–C6 and C6–C7) (1, 2). CSM is a multifactorial disease; and potential genetic, nutritional, morphometric, and kinematic etiologies have been investigated (1, 3). Although not completely clarified, an increased mobility transmitted by the articular facets and a relatively narrow spinal canal combined with a degenerated protruding intervertebral disc (IVD) have been identified as key factors for the development of CSM (1, 3). However, the relative stenosis of the vertebral canal has been questioned in a more recent work (4).

The Doberman Pinscher is a large-breed dog known to be predisposed to CSM. Therefore, this particular breed has been overrepresented in many studies focusing on elucidating the pathogenesis of CSM (5–9). Specific morphometric parameters (e.g., IVD height, vertebral canal size, and foraminal size) of the cervical spine of the Doberman Pinscher have been investigated to obtain further insight into the pathogenesis of CSM (4, 7, 10, 11). Moreover, some of these morphometric characteristics have been compared between disease-free and CSM-affected Doberman Pinschers (5, 9, 12–17). However, no clear difference in terms of morphometric parameters has been identified thus far (4, 5, 10). These inconclusive findings triggered a renovated research enthusiasm on investigating which morphometric parameter may contribute to CMS etiology.

In contrast to medium or large dogs, small-breed dogs are rarely affected by CSM, accounting for 4.8–5% of dogs affected by CSM (18, 19). There is no evidence of CSM in cats. The morphometric differences in the caudal cervical spine of small breeds dogs and cats compared with medium or large dogs is one hypothetical explanation for the difference of CSM prevalence. A recent study investigating the morphometric parameters of the caudal cervical spine of medium-sized dogs, including the IVD wedge angle and IVD width, reported specific morphometric characteristics and significant changes of these parameters, when different loading conditions were applied (20). This study included specimens from mixed-breed dogs weighing 25–35 kg (21–23). In this study, areas of IVD space width were defined as dynamic, if they changed throughout motion, and static, if the IVD space width did not change throughout flexion, extension, or lateral bending. However, it is unclear if these newly described features are different between medium-sized dogs and small dogs and cats. As such, a morphological comparison between dogs commonly affected by CSM and small-breed dogs/cats could generate valuable insights into factors contributing to the development of CSM.

Therefore, the purpose of this study was to investigate the morphometry and kinematic behavior of the caudal cervical spine in small-breed dogs and cats, as it has been done previously in medium-sized dogs (20). We hypothesized that:

- The surface of the caudal vertebral endplates (VEPs) of small-breed dogs and cats is larger than that of the cranial ones, similar to what is seen in medium-sized dogs.
- The VEP surface normalized for body weight is similar between small-breed dogs, medium-sized dogs, and cats.
- The IVD space width in small dogs and cats shows the same distribution of dynamic areas on the VEP as found in medium-sized dogs.

## Materials and Methods

### Sample Population

A total of 11 spinal specimens (C3–Th1) were used. Specimens were collected from five similarly sized small-breed dogs (Jack Russell or similar mixed breed) and six similarly sized cats (domestic shorthair) euthanized due to reasons unrelated to the spinal disease. A written owner consent for donating the animal for research purposes, in agreement with hospital and ethical policy, was obtained for all cadavers. Within 1 h after euthanasia, spinal specimens including surrounding soft tissues were collected, wrapped in saline-soaked towels, and frozen at −20°C for storage purposes. Specimens were stored for 3 months or less prior to testing.

Specimens were thawed for 24 h at 4°C prior to testing. Spinal specimens were cleared of all soft tissues to identify the individual vertebral segments and to reveal potential vertebral abnormalities. The spinal ligaments, IVDs, and joint capsules were left intact.

Specimens were allowed to adapt to room temperature for 4–6 h. Specimens were kept moist during testing using saline spraying or saline-soaked towels whenever possible. Testing was performed under room temperature (~20°C). Prior to testing, radiographs were taken to rule out pathological changes. Specimens were excluded when abnormalities were detected in the radiographs or during specimen preparation including bone lysis, bone proliferation, or calcification in the region of the IVD.

### Specimen Preparation

**Figure 1:**
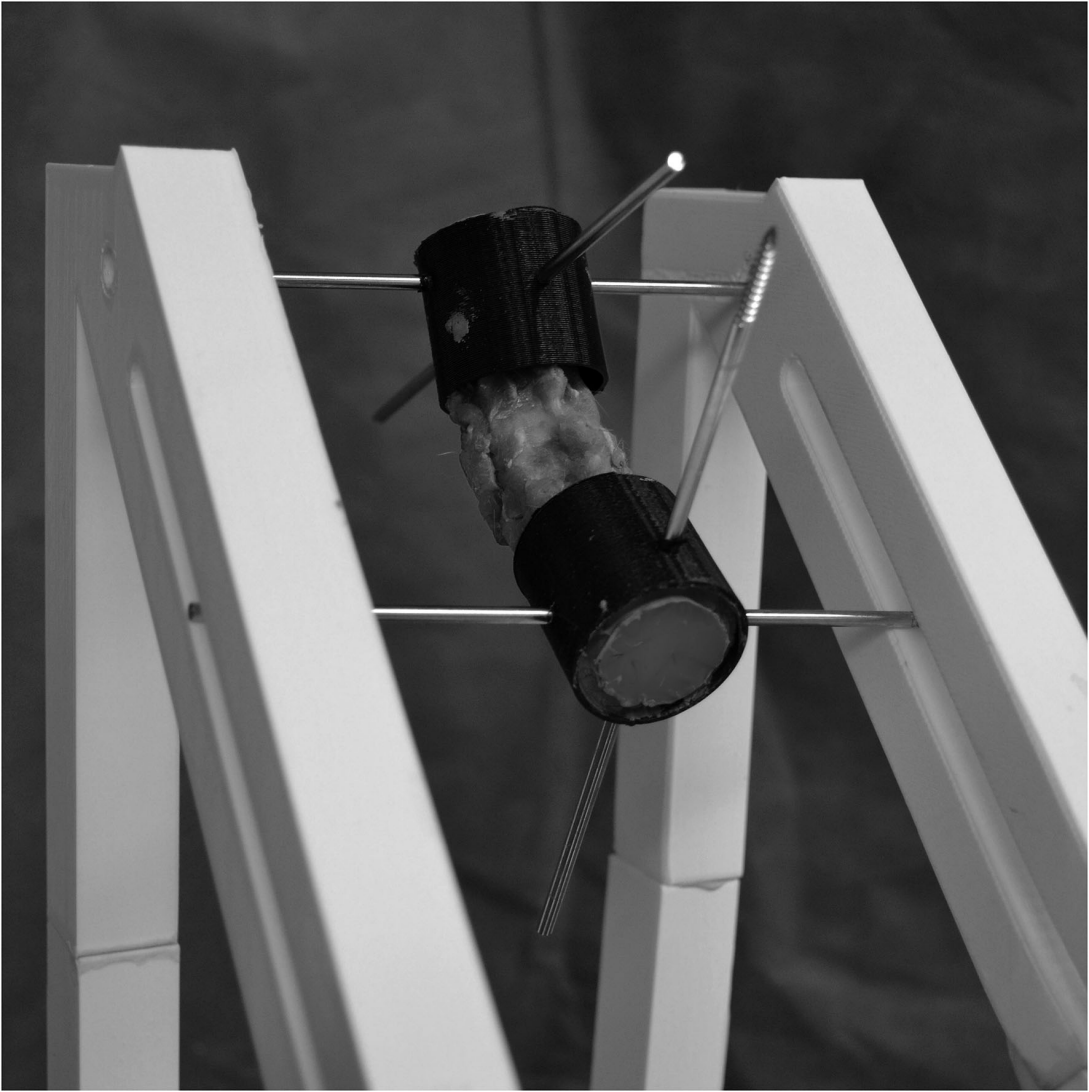
The experimental testing jig for the cervical specimens. Two orthogonal 2.5-mm-diameter Steinman pins were inserted in the transverse and sagittal planes of the potting cylinders. An external skeletal fixator was mounted to maintain different loading positions (neutral, flexion, extension, and lateral bending). Pins in the cranial segment were fixed in a hole in each polyvinylchloride bar, whereas pins in the caudal segment were placed through a slot to direct the applied torque to one direction.

The experimental setup was similar as previously described (20). However, due to the smaller size of the vertebrae, small adaptions to the testing setup had to be made. The spinal specimens used included C3 to Th1. Vertebrae C3–C4 and C7–Th1 were stabilized using K-wires (1.0–1.4 mm), which were inserted axially in the vertebral bodies leaving segments C4–C5, C5–C6, and C6–C7 mobile. Both ends of the spinal specimens were potted into 3D-printed cylinders (30 mm in diameter) using polymethyl methacrylate (PMMA) (Figure 1). The segments C3–C4 and C7–T1 were completely fixed in neutral position. The neutral position was determined with the specimen lying on its lateral side without any force acting on the spine. Before potting, 2.5-mm K-wires were inserted into the cylinders to allow biomechanical loading. One pin was oriented in the transverse plane perpendicular to the vertebral column and pointing in a laterolateral direction, while the other pin was oriented in the median plane in a dorsoventral direction. Inserting the pins into the PMMA rather than into the vertebrae avoided iatrogenic fractures of the vertebrae. Specimens were loaded in a modified 3D-printed loading jig similar as previously used (20). In comparison with the previously used jig, the openings in the 3D-printed blocks were adapted to the smaller size of the K-wires (2.5 mm). Otherwise, the jig was similar to the one used by Knell et al. (20).

### Load Application

A physiologic torque (0.5 N m) was applied to the whole specimen through a handheld scale with the specimen positioned in the jig. The angle of the applied force (as measured between the pulley cable, which was parallel to the ground, and the spinal canal) in relation to the specimen and the lever arm of the applied force were measured to evaluate whether the desired torque of 0.5 N m was applied to each specimen. The applied torque was lower compared with that of previous testing (1.5 N m), as the constructs did not withstand higher loads during pilot testing (20). A load was chosen as higher as possible to be tolerated by both feline and canine specimens, so that the specimens of both groups were loaded under the same conditions. If the load was tolerated, a subjective assessment of the soft tissues and bony structures remaining intact after applying the torque was performed. Specimens were loaded as previously published through cable pulleys and controlled using a handheld scale (20).

The cranial vertebral segment was fixed to the jig by means of the two pins, which then allowed vertebral motion only at the caudal end of a specimen. Each specimen was loaded in flexion, extension, and lateral bending. For flexion and extension, torque was applied to the ventrodorsal pin in the caudal potting cylinder from the dorsal and ventral sides of the vertebra, respectively. Only left lateral bending was performed assuming that right and bending left lateral bending were identical based on previous data (20). After torque application, the specimen was fixed in loading positions using an external skeletal fixator.^1^ The vertebral specimen was then imaged (CT scan; see below) in this position.

### CT Imaging

CT images were obtained from each spinal specimen in the following sequential conditions: (1) native spine (neutral position), (2) spine loaded in flexion, and (3) spine loaded in extension and spine loaded in left lateral bending. CT images^2^ were obtained from each specimen in these positions. The scanning parameters were 120 kV, 250 mA, pitch 0.688, rotation time 0.75 s, and a detector collimation of 16 × 0.75. The raw data were reconstructed in a bone algorithm with an increment of 0.5 mm. CT images were exported to a workstation for data analysis using free DICOM-viewer.^3^

To achieve consistent measurements, the specimens were aligned in the sagittal, dorsal, and transverse planes as previously described (24). In the previous test setup (20), metal beads were inserted in the dorsal spinous process to ensure repetitive alignment. In the current setup, this was not performed because of the small size of the spinal specimens. Instead, alignment was controlled by a point half the height and half the length of the dorsal spinal process. After testing, specimens were dissected to examine them for IVD degeneration (IVDD) according to the Thompson grading scheme (25, 26). Specimens with IVDs with a grade higher than II were excluded from the study.

### Morphometric Variables

Measurements were performed by the primary investigator (SCK) on CT images. Static and dynamic parameters were measured for the IVD spaces C4–C5, C5–C6, and C6–C7. Static variables were defined as measurements that were not changed by vertebral motion, whereas dynamic variables were defined as measurements that changed throughout vertebral motion.

**Figure 2:**
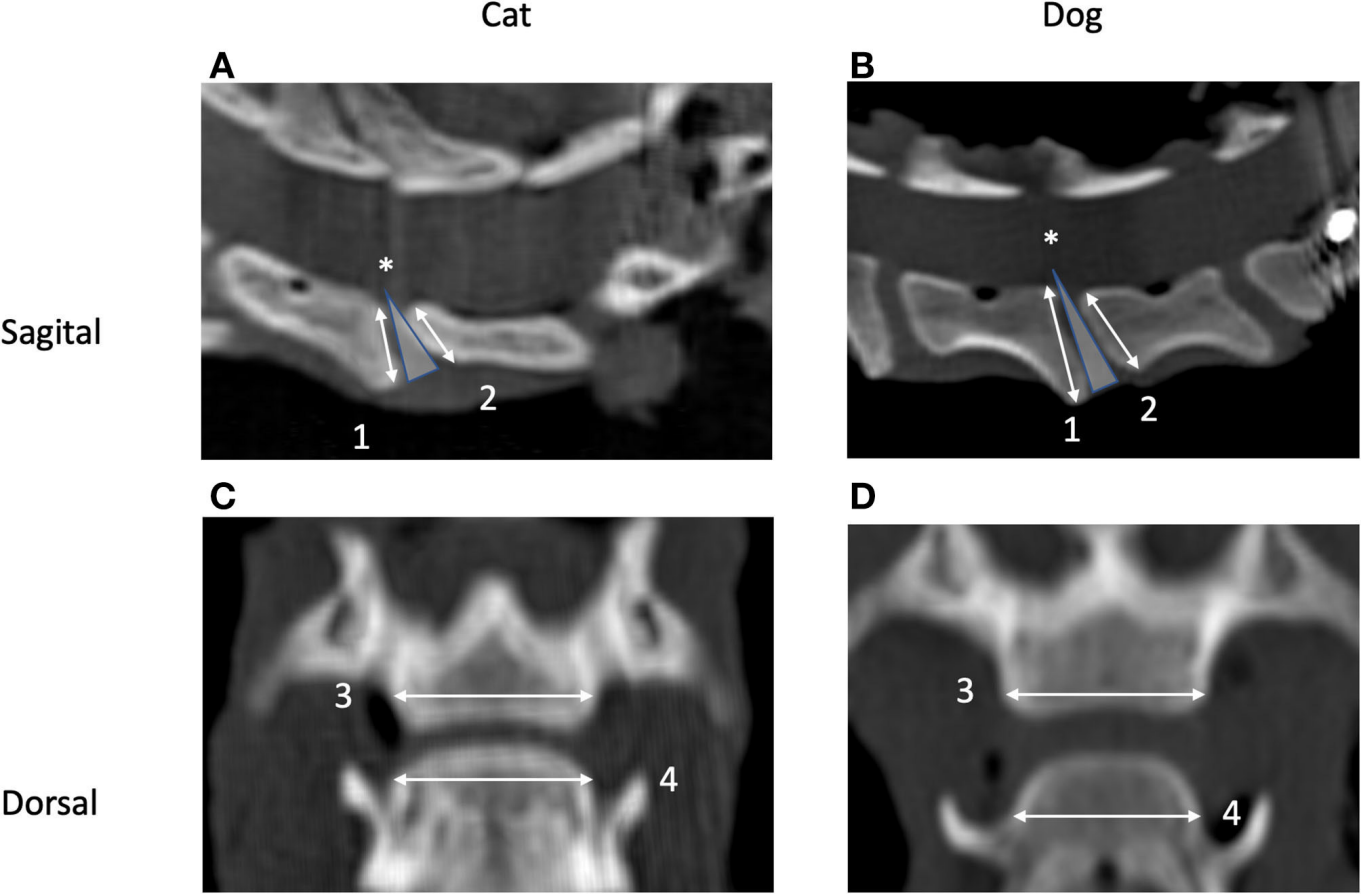
Sagittal **(A,B)** and dorsal **(C,D)** CT images of the segment C5–C6 in a cat **(A,C)** and a dog **(B,D)**. Cranial is to the left **(A,B)** and to the top **(C,D)**. Images originate from the midsagittal **(C,D)** and middorsal planes **(A,B)**. Measurement of height (1 and 2) and width (3 and 4) of the caudal (1 and 3) and cranial (2 and 4) vertebral endplates and definition of the intervertebral disc (IVD) wedge angle (*) are shown as well.

*Static parameters* included the width and height of the cranial and caudal VEPs (Figure 2) and an estimation of the VEP surface. References to a cranial or caudal VEP were made with respect to the cranial and caudal aspects of a particular vertebra and not in relation to the IVD space (e.g., the cranial VEP for the C4–C5 space was the cranial VEP of the vertebral body of C5, and the caudal VEP was the caudal VEP of the vertebral body of C4).

**Figure 3:**
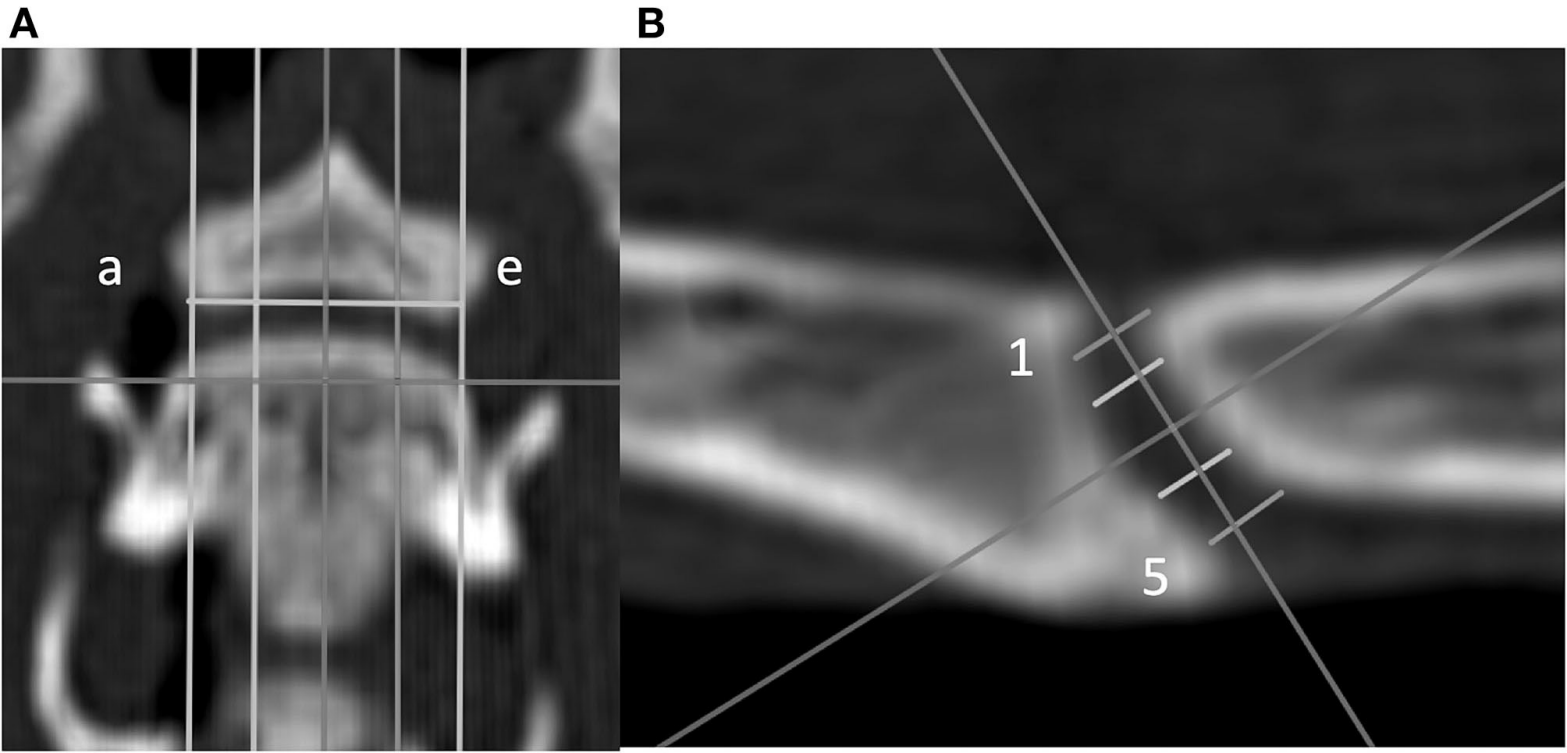
Dorsal **(A)** and sagittal **(B)** CT images of a feline vertebral specimen. **(A)** The image is at the level of the dorsal tip of the caudal vertebral endplate. Lines a through e are equidistant and parallel and divide the intervertebral disc (IVD) space into five sagittal planes. **(B)** Sagittal view corresponding to line c (midline on the dorsal view). Five lines (labeled 1 through 5 from dorsal to ventral) were drawn equidistant and parallel to each other. Measurement of the IVD width was determined for these lines.

- VEP height was defined as the maximum distance from the dorsal to the ventral tip of the VEP as measured in the midsagittal plane (line c in Figure 3A) for both the cranial and caudal VEPs.
- VEP width was defined as the left-to-right margin of the VEP in the middorsal plane (line 3 in Figure 3B).
- VEP height and width were used to approximate VEP surface area, assuming the VEP to have the form of a rectangle. Size was calculated by multiplying height and width. To compare VEP size between the two species, the body weight of the animal was normalized against the VEP surface (body weight ^*^ 9.81/VEP surface). The factor 9.81 was used to convert body weight (kg) into N by the standard gravity. The N/mm^2^ value however must not be misinterpreted as a pressure. The ratio was calculated simply to be able to compare the area in specimens of different body weights. Ratios for medium-sized dogs were similarly calculated from previously unpublished data from a previous study (20).

*Dynamic parameters* included the IVD space width and IVD wedge angle measured in neutral position as well as flexion, extension, and lateral bending. Each of the landmarks was set manually for each CT reconstruction:

- The IVD wedge angle was defined as the angle between two lines that intersected the cranial and caudal VEPs (20). These two lines were defined in the midsagittal plane of the IVD space by the use of the reconstructed CT images. Bony protrusions surrounding the region of the nucleus pulposus of both cranial and caudal VEPs were identified. A line connecting these landmarks was drawn for each VEP. The angle between these two lines was defined as the IVD wedge angle (Figure 2). A positive value indicated that the angle opened ventrally, whereas a negative value indicated that the angle opened dorsally. When these lines were parallel, the angle was recorded as 0°.
- IVD space width was defined as the distance between the cranial and caudal VEPs relying on a grid of 25 landmarks on the VEP as reported previously (20): to create this grid, the first step was to define a dorsal plane at the dorsal tip of the VEP of each segment in the multiplanar CT reconstruction. In this dorsal plane, five equidistant lines were created from the cranial to caudal VEPs parallel to the long axis of the vertebrae from the right lateral to left lateral border of the caudal VEP (lines were labeled a through e from left to right to define five sagittal planes; Figure 3). Each sagittal plane image was exported in DICOM format to enable the measurements using a free DICOM-viewer (see Footnote 3). The IVD space width was measured at five locations and defined separately for each of the five sagittal planes. Line 1 was located between the craniodorsal tip of the cranial VEP and the caudodorsal tip of the caudal VEP. Similarly, line 5 was initiated by drawing a line from the most cranioventral tip of the cranial VEP and the caudoventral tip of the caudal VEP (Figure 3). The additional three lines were added between lines 1 and 5 such that the lines were equidistant. These five lines were labeled from 1 to 5 (dorsal to ventral). By use of lines 1 through 5, IVD width was measured at five dorsoventral levels for all sagittal slices (planes a through e), which resulted in a map of the IVD width at 25 locations. However, because the height of the VEP differed among the sagittal planes (e.g., the VEP height at sagittal plane a was smaller than the VEP height at sagittal plane c), the 25 points on the IVD map were not evenly distributed.
- The range of motion (ROM) was calculated as the difference between the IVD wedge angle in flexion and extension.

### Data Analysis and Statistics

For all variables with continuous data, mean ± SD was calculated separately for the C4–C5, C5–C6, and C6–C7 IVD space width. Changes in IVD width were also expressed as a percentage of the distance from the neutral position. Inferential statistics were performed with a free software.^4^,^5^ Linear mixed models were created to compare static variables between VEP (cranial and caudal), among segments (C4–C5, C5–C6, and C6–C7), between species (dog and cat), and among dynamic variables (IVD space width and ROM) for the four loading positions (neutral, flexion, extension, and lateral bending) and segments. The Akaike information criterion was used for model selection. Normal distribution of the response variables within each model was assessed with PP and QQ plots. The Benjamini–Hochberg correction was used to correct for multiple comparisons. Significance was set at *p* < 0.05.

## Results

### Specimens and Torque Application

For the feline specimens, mean ± SD for body weight and age was 4.0 ± 1.5 kg (range 3.5–5 kg) and 5.3 ± 2.2 years (range 2–9 years), respectively. The body weight and age of the dogs used were 8.0 ± 0.4 kg (range 7.6–8.5 kg) and 10.0 ± 1.7 years (range 7–11 years), respectively. No feline or canine specimen was excluded based on findings during preparation, radiographs, or postmortem evaluation of the IVDs. All cats and 4/5 dogs had IVDs graded as Thompson Grade I. One dog (No. 5) had an IVD (C4–C5) grade as Thompson II, with the remaining IVD grade as Thompson I.

For dogs and cats, respectively, the applied torque was 0.4 ± 0.1 N·m and 0.6 ± 0.2 N·m in flexion, 0.6 ± 0.2 N·m and 0.6 ± 0.2 N·m in extension, and 0.7 ± 0.1 N·m and 0.8 ± 0.1 N·m in lateral bending. Torque was not different between species (*p* = 0.14, *p* = 0.99, and *p* = 0.67 for flexion, extension, and lateral bending, respectively), for any of the loading directions. The increased lever arm due to different potting techniques compared with our previous study was respected in these calculations (20).

### Static Parameters

#### Small-Breed Dog

The mean overall height and width of the caudal VEP were 9.7 ± 0.04 mm and 10.0 ± 0.1 mm, respectively. The mean overall height and width of the cranial VEP were 7.9 ± 0.2 mm and 9.0 ± 0.3 mm among all segments, respectively (Table 1). The caudal VEP was significantly higher (*p* = 0.0009) and wider (*p* = 0.018) than the cranial VEP. No differences were found for VEP height and width between the three spinal segments (*p* = 0.504 and 0.539 for height and width, respectively).

**Table 1:** Dimensions (means ± SD) of the cranial and caudal cervical vertebral endplates in small-breed dogs.

**Variable**	**Endplate**	**C4–C5**	**C5–C6**	**C6–C7**
Height	Caudal	9.7 ± 1.0	10.0 ± 0.9	9.6 ± 0.9
	Cranial	7.4 ± 1.1	8.0 ± 0.7	8.4 ± 0.8
Width	Caudal	10.3 ± 1.2	9.7 ± 1.0	10.1 ± 1.1
	Cranial	8.9 ± 1.2	8.8 ± 0.7	9.3 ± 0.6

The mean surface of the caudal and cranial VEPs was 104.9 ± 17.4 mm^2^ and 76.3 ± 14.5 mm^2^, respectively. The caudal surface was significantly larger than the surface of the cranial VEP (*p* = 0.0008). The relation of body weight to VEP surface was 0.75 ± 0.16 N/mm^2^ and 1.0 ± 0.18 N/mm^2^ for the caudal and cranial VEP, respectively (*p* = 0.0007).

#### Cat

The overall height and width of the caudal VEP were 5.7 ± 1.1 and 6.7 ± 0.7 mm, respectively. The mean overall height and width of the cranial VEP were 5.0 ± 1.8 and 7.3 ± 1.3 mm among all segments, respectively (Table 2). The caudal and cranial VEPs were similar in height (*p* = 0.210), while the caudal VEP was significantly wider (*p* = 0.004) than the cranial VEP. No differences were found for VEP height and width between the three spinal segments (*p* = 0.504 and 0.539 for height and width, respectively).

**Table 2:** Dimensions (mean ± SD) of the cranial and caudal cervical vertebral endplates in cats.

**Variable**	**Endplate**	**C4–C5**	**C5–C6**	**C6–C7**
Height (mm)	Caudal	5.8 ± 1.4	5.6 ± 1.0	5.8 ± 1.1
	Cranial	4.6 ± 2.2	4.9 ± 2.2	5.4 ± 1.0
Width (mm)	Caudal	5.8 ± 1.1	7.3 ± 0.6	7.2 ± 0.7
	Cranial	7.8 ± 1.6	7.1 ± 1.2	7.0 ± 1.0

The mean surface area of the caudal and cranial VEPs was 41.4 ± 5.8 and 31.7 ± 6.2 mm^2^, respectively, with the surface area of the caudal VEP being significantly larger (*p* = 0.0008). The relation of body weight to VEP surface was 0.96 ± 0.13 and 1.2 ± 0.16 N/mm^2^ for the caudal and cranial VEPs, respectively (*p* = 0.0007).

#### Medium-Sized Dogs

The relation of body weight to VEP surface was 0.96 ± 0.88 and 1.37 ± 0.14 N/mm^2^ for the caudal and cranial VEPs, respectively.

#### Comparison of Vertebral Endplate Normalized to Body Weight Including Data From Medium-Sized Dogs

**Figure 4:**
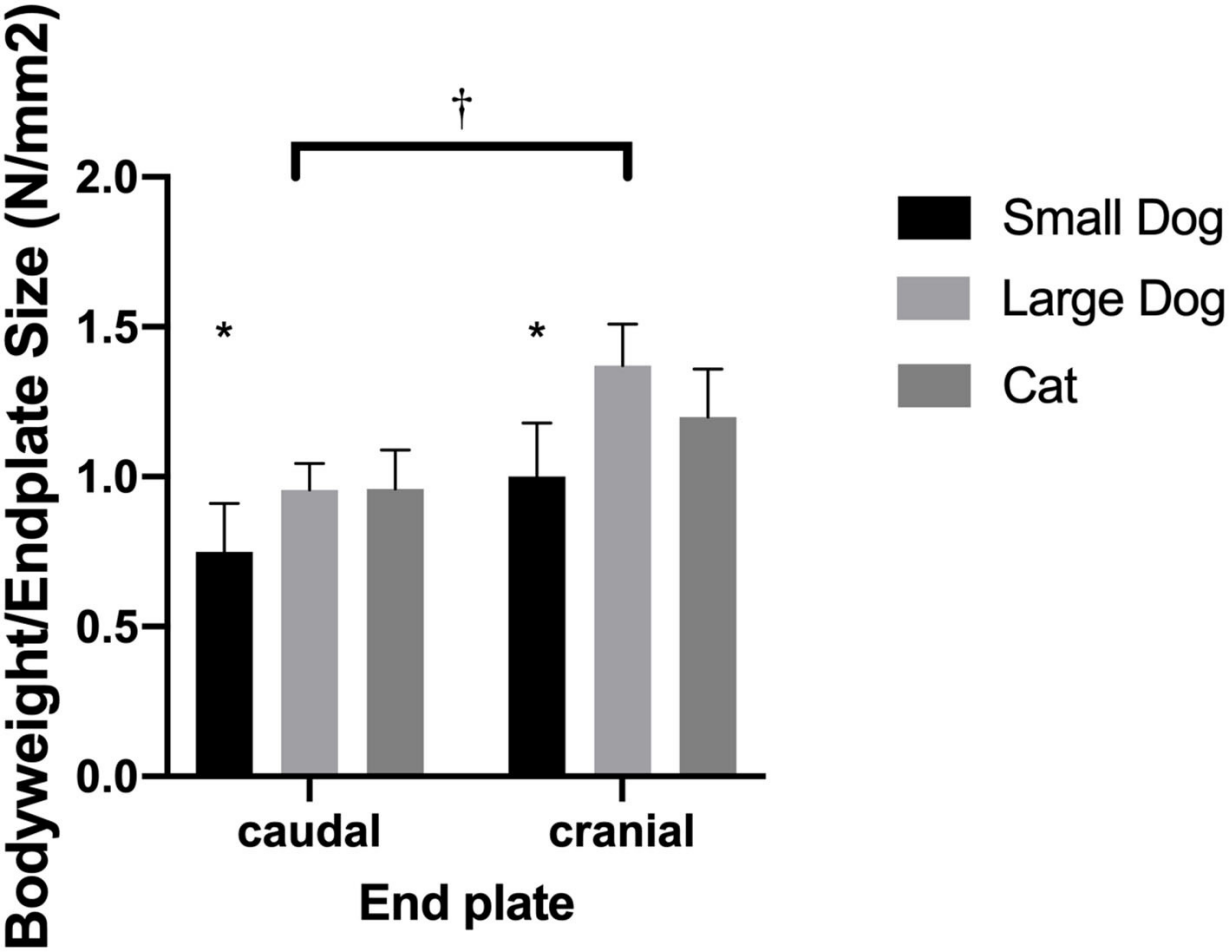
Bar graphs illustrating the different body weight to endplate surface ratio in small- and medium-sized dogs and cats. Results are shown for the caudal and cranial endplate; ratios for the caudal and cranial vertebral endplate were significantly different from each other (^†^). The small dogs showed significantly smaller ratios than cats or medium-sized dogs (^*^).

Normalized surface of the VEPs to the body weight revealed similar (*p* = 0.27) ratios for cats and medium-sized dogs. Small dogs had smaller ratios than cats (*p* = 0.0422) and medium-sized dogs (*p* = 0.0064; Figure 4).

### Dynamic Parameters

#### Small-Breed Dog

**Figure 5:**
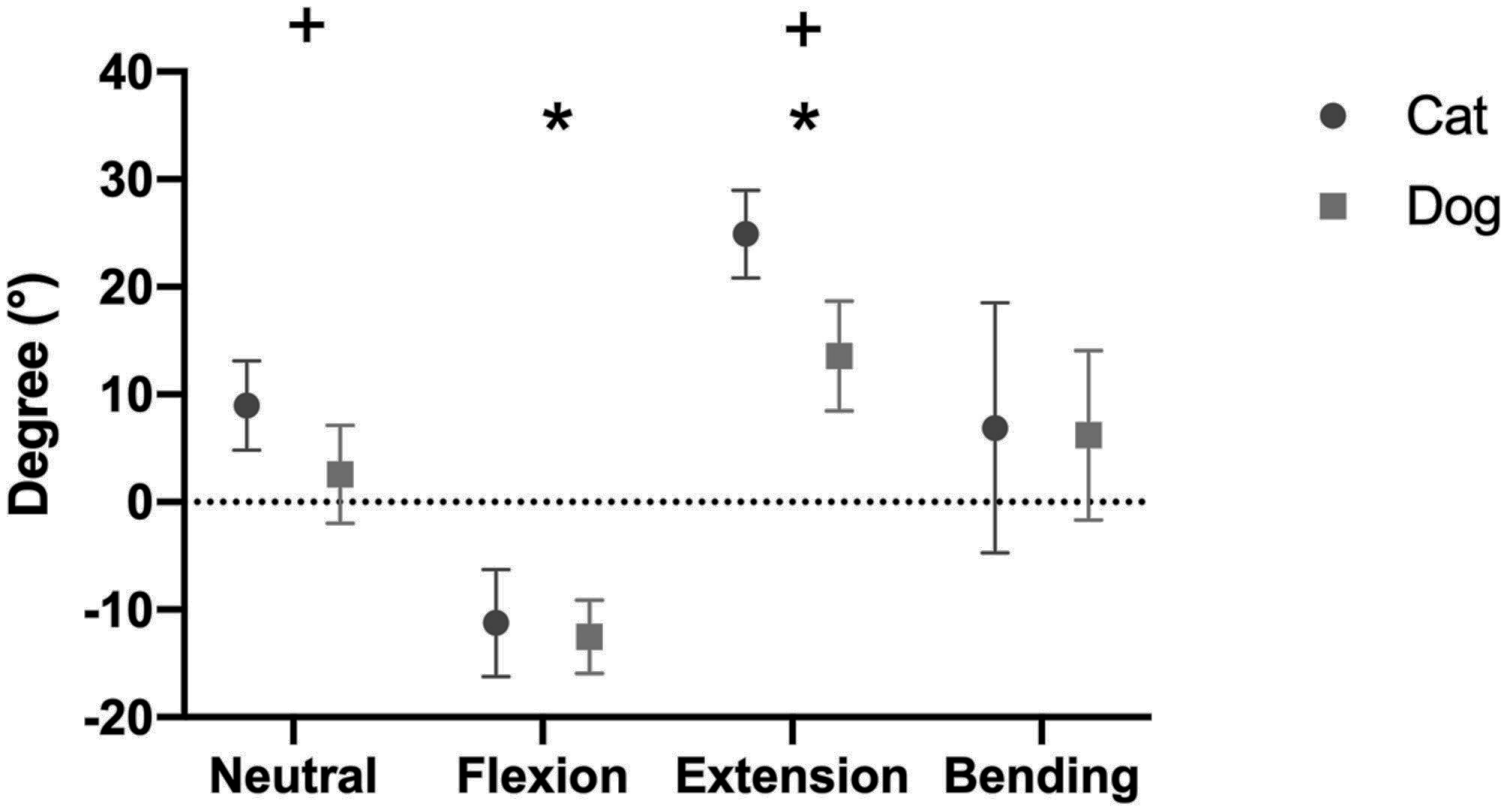
Intervertebral disc (IVD) wedge angles (mean degree ± SD) for the feline and canine spinal specimens in different loading conditions. Canine specimens originated from small dogs. Positive values indicate that the angle opened ventrally, whereas negative values indicate that the angle opened dorsally. *indicates a significant difference between one loading condition and all other loading conditions (*p* < 0.05). ^+^indicates a significant difference between dogs and cats for a specific loading condition (*p* < 0.05).

The overall mean IVD wedge angle in neutral position was 2.6 ± 4.6° among all segments (Table 3). The IVD wedge angle was significantly decreased in flexion (*p* < 0.001) compared with all other loading conditions (Figure 5). The IVD wedge angle was increased significantly in extension compared with the other loading conditions (*p* < 0.001). Lateral bending did not induce a significant change in IVD wedge angle. The total ROM in flexion/extension for all segments was 25.4 ± 7.0°. No differences were found for IVD wedge angle between the three spinal segments (*p* = 0.5292).

**Table 3:** IVD wedge angles (means ± SD) of the different segments and the different motion extremes in small dogs.

**Variable**	**IVD space**	**Neutral**	**Flexion**	**Extension**	**Bending**
Angle (°)	C4–C5	2.2 ± 5.1	−12.3 ± 4.6	13.1 ± 5.4	7.1 ± 4.7
	C5–C6	3.9 ± 5.0	−11.1 ± 2.5	15.0 ± 5.3	10.9 ± 4.1
	C6–C7	1.6 ± 4.3	−14.3 ± 2.7	12.7 ± 5.6	0.6 ± 10.5
	Overall	2.6 ± 4.6	−12.5 ± 3.4	13.6 ± 5.1	6.2 ± 7.9

*IVD, intervertebral disc*.

**Figure 6:**
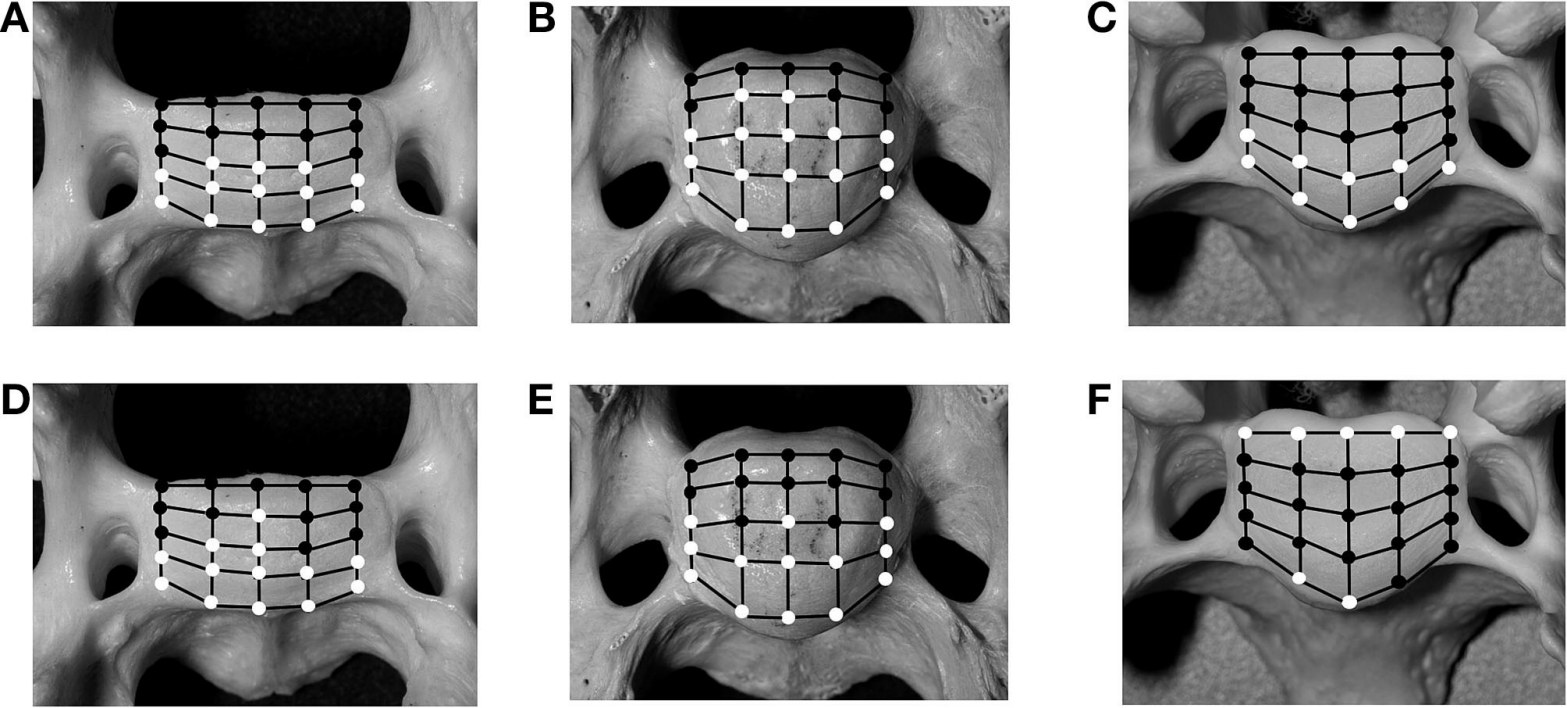
Schematic illustration of 25 predefined landmarks on the cranial bony vertebral endplate of a vertebral specimen from a cat **(A,D)** and a dog **(B,E)**. In comparison, images from a medium-sized dog are shown **(C,F)** as previously published (20). Locations that have significant (*p* < 0.05) changes for intervertebral disc (IVD) measurements in either of the motion extremes [flexion **(A–C)** and extension **(D–F)**], compared with values for the neutral position, are indicated as white circles, whereas locations with non-significant changes are indicated as black circles.

The analysis of the IVD space width revealed an area on the VEP, which changed significantly when different motion extremes were compared (Figure 6). This area represented 68% of the surface of the VEP. The changes of the IVD space width ranged from 1 to 44% (Table 4). In both flexion and extension, changes in IVD space width were seen in the ventral and central areas of the VEP.

**Table 4:** Changes of the IVD space (%) averaged for the segments C4–C5, C5–C6, and C6–C7 width in motion flexion and extension compared with the neutral position in the small dog.

**Motion**	**Line**		**Sagittal plane**			
		**a**	**b**	**c**	**d**	**e**
Flexion	1	16.8	10.4	6.7	16.0	18.2
	2	12.5	**3.9**	**1.4**	0.5	12.6
	3	**2.2**	**−10.5**	**−17.7**	**−16.5**	2.2
	4	**−4.9**	**−40.0**	**−40.1**	**−38.7**	**−6.2**
	5	**−8.0**	**−36.2**	**−42.6**	**−32.0**	**−19.3**
Extension	1	2.7	−12.1	−9.0	−11.4	1.7
	2	4.2	−8.3	−11.7	−14.9	5.4
	3	11.1	11.4	**8.2**	8.3	**9.4**
	4	**14.5**	**36.3**	**44.3**	**36.9**	**13.9**
	5	**12.1**	**33.7**	**30.9**	**33.1**	**15.5**
Bending	1	2.2	−8.1	−4.6	0.96	6.1
	2	5.9	−6.9	−2.3	2.8	7.0
	3	11.2	10.7	17.8	10.1	16.6
	4	8.1	18.3	38.9	30.2	**17.4**
	5	6.9	21.4	**29.8**	**35.4**	17.6

*Significant changes are shown in bold. IVD, intervertebral disc*.

#### Cat

The mean IVD wedge angle in neutral position was 9.0 ± 4.2° among all segments (Table 5). Compared with neutral position, the wedge angle was significantly more positive in flexion (*p* = 0.003) and negative in extension (*p* = 0.002, Figure 5). Lateral bending induced no significant change in IVD wedge angle (*p* = 0.260). The total ROM for all three spinal segments was 35.5 ± 5.8°. No differences were found for IVD wedge angle between the three spinal specimens (*p* = 0.529).

**Table 5:** IVD wedge angles and motion extremes (means ± SD) of the segments C4–C5, C5–C6, and C6–C7 in cats.

**Variable**	**IVD space**	**Neutral**	**Flexion**	**Extension**	**Bending**
Angle (°)	C4–C5	7.9 ± 4.2	−14.4 ± 3.9	24.7 ± 2.1	−1.7 ± 9.2
	C5–C6	8.7 ± 2.5	−12.7 ± 4.1	25 ± 5.4	2.9 ± 10.6
	C6–C7	10.4 ± 5.5	−6.7 ± 3.7	25.1 ± 4.7	12.8 ± 11.6
	Overall	9.0 ± 4.2	−11.3 ± 5.0	24.9 ± 4.1	6.9 ± 11.6

*IVD, intervertebral disc*.

Analysis of the IVD space width revealed an area at the VEP that changed significantly in different motion directions (Figure 6). This area represented 56% of the surface of the IVD. The changes of the IVD space width ranged from 1 to 58.9% (Table 6). In both flexion and extension, changes in IVD space width were seen in the ventral and central areas of the VEP.

**Table 6:** Changes in the IVD space averaged for the segments C4–C5, C5–C6, and C6–C7 width (%) in motion flexion, extension, and lateral bending compared with the neutral position in cats.

**Motion**	**Line**		**Sagittal plane**			
		**a**	**b**	**c**	**d**	**e**
Flexion	1	14.2	18.9	13.2	17.8	14.9
	2	7.1	1.4	−1.1	−1.0	8.4
	3	−13.1	**−20.6**	**−23.6**	**−24.7**	−12.9
	4	**−34.4**	**−40.7**	**−24.2**	**−35.2**	**−35.5**
	5	**−51.7**	**−58.9**	**−49.2**	**−58.1**	**−52.0**
Extension	1	−17.2	−16.7	−12.6	−19.6	−16.6
	2	−8.8	−15.7	**−8.4**	−21.9	−14.4
	3	−3.8	**−2.8**	**−3.9**	−4.1	−13.2
	4	**6.9**	**13.9**	**21.1**	**6.6**	**0.7**
	5	**11.2**	**16.7**	**12.7**	**13.1**	**9.3**
Bending	1	−1.6	−3.4	2.6	−10.4	−7.9
	2	4.8	−4.3	−2.1	−2.9	−6.0
	3	10.0	−3.6	−3.1	−3.7	−4.7
	4	9.7	1.3	−2.6	−5.9	2.4
	5	3.2	−3.4	−13.3	−15.8	−7.9

*Significant changes are shown in bold. IVD, intervertebral disc*.

#### Comparison Between Small Dogs and Cats

The IVD wedge angle was not different between cats and small dogs in flexion (*p* = 0.377), nor in lateral bending (*p* = 0.994). However, cats showed a significantly more positive IVD wedge angle in neutral position (+6.4 ± 0.4°; *p* = 0.0004) and in extension (+11.4 ± 1.0°; *p* = 0.003; Figure 5). The total ROM between flexion and extension was also higher in cats (*p* = 0.0002).

In cats, 56% of the grid locations of the VEP changed the IVD space width when subjected to motion. These locations were located in the ventral and central parts of the VEP, irrespective of the loading direction. In small dogs, 68% of the VEP grid points changed significantly when subjected to motion (Figure 6). This proportion of grid locations changing under loading was not different between small dogs and cats (*p* = 0.56).

## Discussion

The purpose of the current study was to reveal morphometric differences in cervical spinal morphometry between cats and small dogs, generally considered free of CSM disease, to a medium-sized dogs, a subpopulation that can be affected by CSM (20). The current investigation showed that the VEPs and IVD spaces of the caudal cervical spine of small dogs and cats are largely similar with respect to morphometry. The VEP surface of the caudal VEP was larger than the cranial VEPs in both small dogs and cats, leading us to accept our first hypothesis. The VEP size in relation to the body weight was different between small dogs and cats; therefore, we reject our second hypothesis. It was also largely different between small- and medium-sized dogs, which led us to speculate about its role in the pathogenesis of CSM. Finally, we reject our third hypothesis, as the IVD space width in cats and small dogs shows a different distribution of dynamic areas compared with that in medium-sized dogs. The causes of the differences between small dogs and cats and medium-sized dogs identified in this study remain unidentified, and further studies are needed.

### Dynamic Area on the Vertebral Endplate

As previously reported, areas of the VEP can either remain static when the spine is loaded, meaning the IVD space width remains unchanged, or it can change, which has been termed dynamic (20). Looking simply at the number of landmarks on the grid of the VEP that is considered dynamic, the IVD width of the small-breed dogs and cats shows no difference compared with that of medium-sized dogs (20). However, based on the results found in the present study, there is a difference with respect to the dynamic area over the surface of the VEP: in cats and small dogs, only the ventral aspect of the annulus is dynamic in both flexion and extension, with the IVD space width decreasing in flexion and increasing in extension. The dorsal annulus remains static, with no significant changes found in IVD space width through the different motion directions. In contrast, in medium-sized dogs, the IVD space width changes significantly in the region of the dorsal and ventral annuli, but not the central region of the VEP (i.e., nucleus pulposus) (20).

The lower mobility of the dorsal region of the IVD may be a protective factor against dorsal annulus degeneration in the spinal region in small dogs and cats. Excessive motion has been suggested as one potential factor to contribute to IVDD (27–29). It may be speculated that the differences between our results and our previously reported data support the theory that increased mobility at the IVD joint contribute to degenerative IVD disease of the caudal cervical spine in medium-sized dogs (20). However, to test this hypothesis, histopathological and biochemical investigations comparing the IVDs of small-breed dogs, cats, and medium-sized dogs are necessary.

### Relation of Vertebral Endplate Surface to Body Weight

The body weight was normalized to the relative surface area of the highest VEP in the medium-sized dogs, even though this difference was not significant between cats and medium-sized dogs. However, this means that load at the VEP is distributed over a smaller surface leading to an increased pressure in larger dogs. As increased pressure is considered a potential initiation of disc degeneration, this might be a potential explanation for CSM in these dogs (30–32). CSM is considered multifactorial, meaning that most likely the surface size of the VEP and the consecutive increased absolute pressure is contributing to the development of the disease (30–32).

### Wedge Angle

The IVD wedge angle is an angle indicating the orientation between the cranial and caudal VEPs. In human medicine, the IVD wedge angle is used to specify vertebral morphometry with the aim to design IVD disc prostheses and to measure the mobility of the vertebrae after disc arthroplasty (33, 34). The present study showed that cats have a more positive IVD wedge angle (9.0 ± 4.2°) than small (2.6 ± 4.6°) and medium-sized dogs (5.0 ± 2.6°) (20). It remains unclear if the wedge angle is related to the mobility of the spine or plays a role in the pathogenesis of CSM. Looking at the ROM in flexion and extension (calculated on the basis of the IVD wedge angle), the feline spine is more mobile in the sagittal plane (31 to 38°) than that of dogs (ROM < 20°) (20). This finding is supported by the subjective clinical finding that a cat is more mobile than a dog.

### Relation to Previous Study

The current study investigated segments C4–C5, C5–C6, and C6–C7 of small-breed dogs and cats under similar conditions as previously published (20). The investigated specimen originated from breeds or species free or rarely affected by CSM (18, 19). In contrast, our previous study investigated dogs that potentially are affected by CSM. Although the Doberman was not included in that study, recent studies indicate that mixed-breed dogs or breeds other than the Doberman with a body weight between 25 and 35 kg are commonly affected by CSM (21, 23, 35). Therefore, we believe that our previous study represents a group of healthy medium-sized dogs that may belong to a canine population that may be affected by CSM. Contrariwise, the small dogs and cats investigated in this study belong to a population that is rarely affected by CSM.

### Comparison Between Small Dogs and Cats

In this study, two different species were examined: one (cat) completely free of CSM and the other one (small dog) showing a very low incidence of CSM (18, 19). When IVDD was reviewed in two large groups of dogs, the more frequently occurring cervical site was the cranial one, and the Jack Russell was not in the breeds that commonly presented with IVDD (36, 37). However, the type of IVDD was not taken into consideration. Within these two species, we chose to use specimens of similar breeds: domestic short-haired cats and Jack Russell or Jack Russell mixed-breed dogs. These groups were chosen to have very similar groups but on the other hand only allow to conclude on these. We found differences like the increased ROM in cats or the relation between body weight and endplate surface, but also similarities like the static behavior of the dorsal annulus. We did not show if they are related to the pathogenesis of CSM, but we suppose that having two “control groups” makes the analysis of our data more reliable in relation to the pathogenesis of CSM. However, based on our results, we cannot make advance conclusions about CSM pathogenesis. It appears controversial that an increased ROM, which has been related to CSM, does not cause CSM in cats (38). Similarities to another disease-free group, like the static behavior of the annulus fibrosus, might help to filter important findings of the study in regard to the pathogenesis of CSM.

### Limitations

One of the limitations of the present study is the *ex vivo* nature, which reduces the clinical relevance due to the inability to simulate the complex *in vivo* spine motion. The custom-made jig might have constrained the normal physiologic kinematics of the spinal specimens. Therefore, the dynamic parameter values presented in this study may not directly represent the *in vivo* values. However, we believe that the current study setup was adequate to test the research hypotheses posed in this study.

Secondly, as the feline specimens did not withstand a torque of 1.5 N m, the load was reduced to target a torque 0.5 N m. The load was also applied through two immobilized segments, which were respected in the calculation of the total torque for each specimen. This makes the results from the current study not directly comparable with the results obtained for medium-sized dogs using a similar setup, as both setups only allowed to target a torque rather than precise application (20). However, despite the reduction in torque, the spinal specimens were loaded to their extremes. In addition, the degree to which the specimens were loaded, as measured e.g., by the ROM, was comparable with what was observed in medium-sized dogs. Therefore, we believe that the testing conditions are largely similar and hence comparable between the two studies. Lastly, using the described setup, no preload, associated with the weight of the head, was applied to the spinal specimens. This preload has been described in both bi- and quadrupeds as the standard experimental setup to mimic the axial compression, which is believed to have a significant behavior on IVD biomechanics (39–41). However, the feline spine is particularly cumbersome because of the inherent flexibility and predefined shape, making it in our setup impossible to apply an axial preload.

### Conclusion

The morphometry of the cranial and caudal VEPs of small dogs and cats appears largely similar. Small dogs and cats have a similar distribution of dynamic areas within the IVD space. However, this distribution is different from what has been reported for medium-sized dogs with higher mobility at the dorsal region of the VEP. The role of these differences in the development of CSM requires further investigation.

## Data Availability Statement

The original contributions presented in the study are included in the article/Supplementary Files, further inquiries can be directed to the corresponding author/s.

## Ethics Statement

Ethical review and approval was not required for the animal study because Tissue used in this study was harvested from cadavers. Therefore, according to Swiss law, no ethical approval is necessary. Written informed consent was obtained from the owners for the participation of their animals in this study.

## Author Contributions

SK wrote the majority of the manuscript. SK, LS, and AP designed the study. SK performed the experiments including all CT measurements. SK and LS analyzed all data. LS performed the statistical analysis. All authors took part in editing the manuscript.

## Conflict of Interest

The authors declare that the research was conducted in the absence of any commercial or financial relationships that could be construed as a potential conflict of interest.

## Publisher's Note

All claims expressed in this article are solely those of the authors and do not necessarily represent those of their affiliated organizations, or those of the publisher, the editors and the reviewers. Any product that may be evaluated in this article, or claim that may be made by its manufacturer, is not guaranteed or endorsed by the publisher.

## Footnotes

^1^Kirschner–Ehmer apparatus, DePuy Synthes, Oberdorf, Switzerland.

^2^Brilliance CT 16-slice machine, Philips AG, Zurich, Switzerland.

^3^Horos Imaging Software v.1.1.7, RRID:SCR_017340.

^4^R Project for Statistical Computing, RRID:SCR_001905.

^5^GraphPad Prism, RRID:SCR_002798.
