# Supplementary material for: Ex vivo Evaluation of the Dynamic Morphometry of the Caudal Cervical Intervertebral Disc Spaces of Small Dogs and Cats
Source: Front Vet Sci. 2021 Aug 16;8:706452. doi: 10.3389/fvets.2021.706452 (PMC8415525; doi:10.3389/fvets.2021.706452)
Supplement: Supplementary file 1 [file Data_Sheet_1.docx]

Supplementary Material

# Supplementary Figures and Tables

**Table 1:** Table showing the coordinates (a1 to e5) of the grid imposed on the vertebral end plate of the *canine* cervical vertebra. For each coordinate, P values are noted and highlighted (italics) if significant.

Neutral vs Flexion

|  | **a** | **b** | **c** | **d** | **e** |
| --- | --- | --- | --- | --- | --- |
| **1** | 0.95 | 0.95 | 0.21 | 0.93 | 0.59 |
| **2** | 0.42 | *0.027* | *0.0269* | 0.051 | 0.41 |
| **3** | *0.017* | *0.0165* | *0.0006* | *0.0006* | *0.02* |
| **4** | 0.0007 | *0.0007* | *0.0006* | *0.006* | *0.0006* |
| **5** | *0.001* | *0.0001* | *0.0006* | *0.006* | *0.0005* |

Neutral vs Extension

|  | **a** | **b** | **c** | **d** | **e** |
| --- | --- | --- | --- | --- | --- |
| **1** | 0.26 | 0.26 | 0.75 | 0.32 | 0.87 |
| **2** | 0.53 | 0.77 | 0.73 | 0.86 | 0.59 |
| **3** | 0.042 | 0.06 | *0.039* | 0.06 | *0.0451* |
| **4** | *0.0005* | *0.0005* | *0.0005* | *0.0005* | *0.0005* |
| **5** | *0.0003* | *0.0005* | *0.0005* | *0.0005* | *0.0005* |

Neutral vs lateral Bending

|  | **a** | **b** | **c** | **d** | **e** |
| --- | --- | --- | --- | --- | --- |
| **1** | 0.24 | 0.71 | 0.95 | 0.97 | 0.38 |
| **2** | 0.46 | 0.90 | 0.79 | 0.74 | 0.19 |
| **3** | 0.21 | 0.42 | 0.35 | 0.38 | 0.07 |
| **4** | 0.10 | 0.08 | 0.05 | 0.0514 | *0.04* |
| **5** | 0.32 | 0.07 | *0.0084* | *0.011* | 0.14 |

**Table 2:** Overview of P values for the individual coordinates (a1 to e5) of the grid imposed on the vertebral end plate of the *feline* cervical vertebra. Significant P-values have been highlighted in italic text.

Neutral vs Flexion

|  | **a** | **b** | **c** | **d** | **e** |
| --- | --- | --- | --- | --- | --- |
| **1** | 0.99 | 0.99 | 0.08 | 0.82 | 0.051 |
| **2** | 0.88 | 0.37 | 0.42 | 0.36 | 0.60 |
| **3** | 0.14 | *0.036* | *0.011* | *0.017* | 0.064 |
| **4** | *0.0004* | *0.001* | *0.023* | *0.0013* | *0.0002* |
| **5** | *0.0003* | *0.0003* | *0.0004* | *0.0003* | *0.0002* |

Neutral vs Extension

|  | **a** | **b** | **c** | **d** | **e** |
| --- | --- | --- | --- | --- | --- |
| **1** | 0.99 | 0.46 | 0.18 | 0.59 | 0.94 |
| **2** | 0.21 | 0.37 | *0.0028* | 0.62 | 0.56 |
| **3** | 0.06 | *0.044* | *0.0063* | 0.058 | 0.46 |
| **4** | *0.0016* | *0.0004* | *0.0002* | *0.003* | *0.017* |
| **5** | *0.0016* | *0.0004* | *0.0002* | *0.0002* | *0.0002* |

Neutral vs lateral Bending

|  | **a** | **b** | **c** | **d** | **e** |
| --- | --- | --- | --- | --- | --- |
| **1** | 0.99 | 0.99 | 0.89 | 0.81 | 0.75 |
| **2** | 0.78 | 0.99 | 0.99 | 1.00 | 0.87 |
| **3** | 0.59 | 0.98 | 0.99 | 0.99 | 0.93 |
| **4** | 0.59 | 0.92 | 1.00 | 0.93 | 0.86 |
| **5** | 0.81 | 0.95 | 0.56 | 0.38 | 0.74 |
